# Electrospinning of Polycaprolactone Membranes Using Green Solvents for Organ-on-a-Chip Applications

**DOI:** 10.3390/polym18121547

**Published:** 2026-06-22

**Authors:** Donna Danijela Dragun, Karla Kuzman, Marta Blažek, Petra Popović, Floren Radovanović-Perić, Iva Rezić Meštrović, Fabio Faraguna, Ernest Meštrović

**Affiliations:** 1 Faculty of Chemical Engineering and Technology, University of Zagreb, Trg Marka Marulića 19, 10000 Zagreb, Croatia; ddragun@fkit.unizg.hr (D.D.D.); ppopovic@fkit.unizg.hr (P.P.); fradovano@fkit.unizg.hr (F.R.-P.); ffaragun@fkit.unizg.hr (F.F.); 2 Faculty of Textile Technology, University of Zagreb, Prilaz Baruna Filipovića 28a, 10000 Zagreb, Croatia; iva_rezic@net.hr

**Keywords:** acetic acid, Design of Experiments, electrospinning, green solvents, nanofibers, organ-on-a-chip, polycaprolactone, tissue engineering

## Abstract

Electrospinning is a highly versatile technique for fabricating nanofibrous membranes with high surface-area-to-volume ratios and tunable porosity. Although polycaprolactone (PCL) is widely utilized in biomedical engineering due to its biocompatibility, its electrospinning traditionally relies on hazardous organic solvents like dichloromethane (DCM) and N,N-dimethylformamide (DMF). This paper details the development of a fully sustainable, green electrospinning process for PCL using a bio-derived binary mixture of acetic acid and formic acid. Processing parameters (applied voltage, tip-to-collector distance, and flow rate) were systematically optimized using a Design of Experiments (DoE) response surface methodology. Scanning electron microscopy (SEM) confirmed the successful fabrication of uniform, bead-free nanofibers with a mean diameter of 247 nm, representing a 37.3% reduction compared to conventional DCM:DMF-spun matrices. Fourier-transform infrared spectroscopy (FTIR) verified complete solvent evaporates.

## 1. Introduction

Organ-on-a-chip (OoC) technology has emerged as one of the most promising paradigms in biomedical research and pharmaceutical development, offering a physiologically relevant, three-dimensional microenvironment that recapitulates the structural and functional complexity of human tissues and organs [1]. These micro-engineered systems integrate living cells within precisely defined microfluidic channels and scaffolding materials, enabling the dynamic simulation of tissue interfaces such as the gut epithelium, blood–brain barrier, lung alveoli, and vascular endothelium. A key driver behind the growing adoption of OoC platforms is the imperative to reduce and, ultimately, replace animal models in preclinical research. OoC models are developing significantly, and the current state of the art includes “heart-on-a-chip”, “liver-on-a-chip”, “brain-on-a-chip”, and “intestine-on-a-chip” as either single-organ models or multi-organ models. However, despite groundbreaking progress in OoC systems, there is a growing need for monitoring tissue/organ functions and microenvironments on-a-chip to promote large-scale and automated applications [2]. The 3Rs framework (Replacement, Reduction, Refinement) has been formally embedded in U.S. drug development legislation through the FDA Modernization Act 2.0 (Public Law 117-328, signed December 2022), which for the first time explicitly authorizes the use of non-animal new approach methodologies (NAMs)—including micro-physiological systems, organoids, and computational models—as alternatives to animal studies in support of Investigational New Drug (IND) applications and Biologics License Applications (BLAs) [3,4,5].

Building on this legislative mandate, the FDA launched its Innovative Science and Technology Approaches for New Drugs (ISTAND) Pilot Program as a qualification pathway for drug development tools that fall outside the scope of existing programs. In September 2024, the ISTAND Program accepted the first organ-on-a-chip submission: a human Liver-Chip designed to predict drug-induced liver injury (DILI), which correctly identified 87% of hepatotoxic drugs in a blinded validation study [6]. In parallel, the FDA published its “Roadmap to Reducing Animal Testing in Preclinical Safety Studies” (2025), setting up a 3- to 5-year horizon to make animal studies the exception rather than the norm [7]. A comprehensive review by the U.S. Government Accountability Office (GAO-25-107335, 2025) further underscored the strategic importance of OoC technology while identifying key challenges including standardization and cross-platform validation [8].

Central to the function of OoC devices is the choice of scaffold material, which must serve as a mechanically stable, biocompatible, and biologically inert substrate for cell attachment, proliferation, and differentiation [9,10]. Electrospun fibrous membranes are particularly well suited for this role due to their structural resemblance to the native extracellular matrix (ECM), their high porosity facilitating nutrient and gas transport, and the tunability of fiber diameter, alignment, and surface chemistry [11,12].

Polycaprolactone (PCL, CAS 24980-41-4) is a semicrystalline, hydrophobic polyester with a low melting point (~60 °C), excellent mechanical ductility, and a controllable degradation rate spanning months to years depending on molecular weight and crystallinity. These attributes make PCL one of the most extensively investigated polymers for tissue engineering scaffolds and drug-delivery matrices [12]. It has received regulatory approval (FDA, CE marking) for use in a variety of implantable medical devices [13,14,15]. Despite its attractive properties, the conventional electrospinning of PCL almost invariably employs toxic organic solvents, most notably N,N-dimethylformamide (DMF) and dichloromethane (DCM). Both solvents are classified as substances of very high concern (SVHCs) by the European Chemicals Agency (ECHA) under the REACH regulation. DMF is a known reproductive toxicant and suspected human carcinogen, while DCM is a probable human carcinogen with the significant inhalation of hazards. Residual solvent contamination in electrospun scaffolds intended for cell culture or implantation represents a critical safety concern that is difficult to eliminate entirely without compromising fiber morphology [16,17].

The principles of green chemistry, as codified by Anastas and Warner in their twelve principles, mandate the preference for safer solvents and auxiliaries wherever technically feasible. Acetic acid (glacial, CH_3_COOH) and formic acid (HCOOH) are two bio-derived, readily biodegradable organic acids that have attracted attention as greener alternatives for dissolving polyesters [18,19]. Both are listed among the preferred or acceptable solvents in pharmaceutical solvent selection guides (e.g., CHEM21, ICH Q3C), and their vapor phase toxicity profiles are substantially more favorable than those of DMF or DCM. Binary mixtures of acetic and formic acid can modulate the dielectric constant and viscosity of the spinning dope, thereby influencing the electrospinning jet stability and ultimate fiber morphology [20,21].

The systematic optimization of electrospinning parameters is essential to achieve reproducible, bead-free fibrous membranes [22,23,24]. Design of Experiments (DoE) methodology allows efficient exploration of the multidimensional parameter space (polymer concentration, applied voltage, flow rate, needle-to-collector distance, solvent composition) while minimizing the number of experimental runs required. Response surface methodology (RSM) applied to DoE data enables the construction of predictive mathematical models that quantify factor interactions and identify optimal operating conditions [22].

Nanolayered materials can be characterized using a wide range of analytical techniques. The surface characterization of coated samples involves a comprehensive approach employing several complementary methods. Techniques such as X-ray photoelectron spectroscopy (XPS), Raman spectroscopy (RS), potentiometric titration, and atomic force spectroscopy (AFS) are commonly applied to investigate the surface structure and elemental composition. In addition, determination of the chemical composition is supported by methods including energy-dispersive X-ray spectroscopy (EDS), inductively coupled plasma optical emission spectrometry (ICP-OES), inductively coupled plasma mass spectrometry (ICP-MS), secondary ion mass spectrometry (SIMS), and related analytical techniques [25]. After characterization, the application of novel nanomembranes can be further optimized [26,27]. However, this additional step requires careful engineering to ensure efficient incorporation and controlled functionality [28,29,30].

This paper reports on the fabrication of PCL nanofibrous membranes by electrospinning from acetic acid/formic acid solutions and the structural characterization of the resulting membranes by SEM, DSC, TGA, X-ray diffraction and FTIR [14,20]. However, according to our knowledge, this is the first attempt to perform the systematic DoE-guided optimization of processing conditions and to predict and optimize the size of the electrospun samples. Moreover, the hypothesis of this paper was that electrospinning with green solvents is not only environmentally friendly but also enables achieving mechanical properties better than by using toxic solvents (e.g., thinner nanofibers and smaller number of beads).

The suitability of the obtained membranes as cell-scaffolding substrates in OoC systems—including multi-sensor platforms and multi-layered tissue constructs relevant to next-generation pharmaceutical testing and drug formulation development, are discussed in the context of current biomedical and regulatory requirements [23].

## 2. Materials and Methods

### 2.1. Materials

Polycaprolactone (PCL, *M_n_* 80,000 g/mol, Sigma-Aldrich, Darmstadt, Germany, Europe) was used as received. Glacial acetic acid (≥99.7%, Kemika, Zagreb, Croatia, Europe) and formic acid (≥98%, Kemika, Zagreb, Croatia, Europe) served as green solvents. Dichloromethane (DCM) was purchased from CARLO ERBA Reagents, S.A.S, Val de Reuil, France, Europe, and dimethyl formamide, DFM from GRAM-MOL d.o.o., Zagreb, Croatia, Europe. All chemicals were used without further purification. Ultrapure water (resistivity 18.2 MΩ·cm, Milli-Q^®^, Merck, Darmstadt, Germany, Europe) was used where aqueous solutions were required.

### 2.2. Preparation of Spinning Solutions

PCL solutions were prepared by dissolving the polymer in binary solvent mixtures of acetic acid and formic acid at predetermined ratios, which were selected based on our preliminary research. The mixtures were stirred at room temperature for 24 h until complete dissolution was achieved and optically clear, homogeneous solutions were obtained. Polycaprolactone concentrations ranged from 10 to 20% (*w*/*v*). Solvent volumetric ratios of acetic acid to formic acid were varied as specified by the DoE design matrix (Section 2.4), in which the PCL concentrations were kept constant at 12% (*w*/*v*).

### 2.3. Electrospinning Setup

Electrospinning was carried out using an in-house-built laboratory-scale single-needle electrospinning apparatus equipped with a high-voltage power supply (Genvolt, New Road, Shropshire, UK), a precision syringe pump (Kd Scientific, LEGATO 210, Harvard Bioscience Inc., Holliston, MA, USA), and a grounded flat aluminum foil collector. A blunt-tipped stainless steel needle of gauge 21 G was used. All experiments were conducted under controlled ambient conditions: temperature 23 ± 2 °C, relative humidity 40 ± 5%. The following parameters were varied within the ranges defined by the DoE: applied voltage (8–17 kV), tip-to-collector distance (8–17 cm), and flow rate (0.10–1.7 mL/h). Fibers were collected on the aluminum foil collector for a duration of 30 min per run to ensure a uniform membrane thickness.

### 2.4. Design of Experiments

A Central Composite Design (CCD) was implemented using Design-Expert software (Stat-Ease Inc., Minneapolis, MN, USA; version 9.1) to investigate the effects of the following independent variables on fiber morphology: (i) applied voltage (kV), (ii) flow rate (mL/h), and (iii) tip-to-collector distance (cm) (Table 1). The primary response variable was mean fiber diameter (nm) as determined by SEM image analysis. Secondary responses included fiber diameter distribution (standard deviation) and the absence/presence of beads. The experimental design comprised a total of 20 experimental runs, including center-point replicates to estimate pure experimental error and assess model reproducibility.

### 2.5. Electrospun Fiber Characterization

Membranes were investigated in preliminary experiments by optical microscope Olympus B50 (Olympus Europa, Hamburg, Germany). The morphological characterization of electrospun membranes was performed by scanning electron microscopy using a Tescan Vega III Easyprobe (Brno, Czech Republic). Prior to imaging, membrane samples were sputter-coated with a thin layer of gold/palladium alloy under an argon atmosphere to prevent charging. Images were acquired at accelerating voltages of 5–10 kV. Fiber diameters were determined by the measurement of at least 100 fibers per sample using ImageJ software (v. 1.54, NIH, Bethesda, MD, USA), and results are reported as mean ± standard deviation.

The FTIR spectra of PCL material and electrospun membranes were recorded using a FTIR spectrometer (SHIMADZU, IRTracer-100, Tokyo, Japan) equipped with an attenuated total reflectance (ATR) accessory (diamond crystal). Spectra were collected in the range 4000–400 cm^−1^ with a resolution of 4 cm^−1^ and 32 scans per spectrum. Background correction was performed prior to each measurement. Peak identification was carried out using standard PCL reference spectra and literature data.

Differential scanning calorimetry of the PCL material and electrospun membranes was recorded using a Mettler Toledo DSC 3 (Zurich, Switzerland) in nitrogen atmosphere. The samples were subjected to multiple thermal cycles as follows: cooling from 25 to −70 °C, heating from −70 to 160 °C, cooling from 160 to −70 °C and finally, heating from −70 to 200 °C with the temperature rate of 10 °C min^−1^. Thermo-gravimetric analysis was performed on Mettler Toledo TGA/DSC 3+ (Zurich, Switzerland) in nitrogen from 25 to 700 °C. The data obtained were analyzed using the Mettler Toledo STARe software version 18 where enthalpies and thermal transitions were determined.

The crystal phase and crystallinity were examined by X-ray diffraction using a Bruker D8 Advance (Billerica, MA, USA) diffractometer in Bragg–Brentano configuration. A copper cathode (8.04 keV) at 25 mA and 40 kV was used to measure the patterns which were collected with a 0.02° step with a retention time of 0.8 s in the 5–70 2θ range.

Tensile tests were performed on a TA Instruments HR30 (Newcastle, DE, USA) rheometer with axial geometry used for tension tests. Sample size was as follows: width—10 mm, height—20 mm, thickness—100 micrometers. The samples were prepared by cutting the membranes which were still adhered to the aluminum foil which was removed after the sample was loaded onto the device and slightly stretched with a force of 0.1 N. Tensile tests were performed at a 10 µms^−1^ rate until breakage. A total of three measurements were performed for each sample. To obtain the comparable results for the reference, a 150 µm foil was hot pressed from the raw PCL beads.

### 2.6. Statistical Analysis

All quantitative data are expressed as the mean ± standard deviation (SD) of at least three independent experiments unless otherwise stated. Analysis of variance (ANOVA) was applied to DoE response data, and factor significance was assessed at a 95% confidence level (*p* < 0.05). Model adequacy was evaluated using the coefficient of determination (*R*^2^ and adjusted *R*^2^) and lack-of-fit tests.

## 3. Results and Discussion

### 3.1. Solution Properties and Electrospinnability

**Figure 1:**
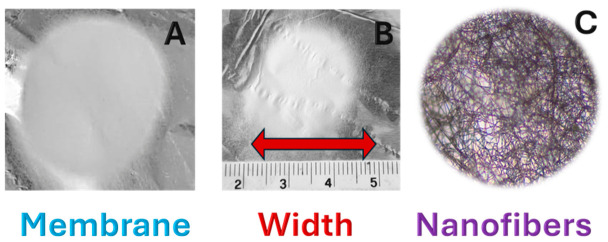
Membrane produced by electrospinning: (**A**) membrane (**B**) measurement of the height and the width of the sample membrane, (**C**) micrograph of electrospun membrane obtained by optical microscopy (Olympus B50) under magnification of 100 times.

PCL dissolved readily in all tested acetic acid/formic acid mixtures at room temperature without heating, which is a significant practical advantage over processes requiring elevated temperatures or prolonged stirring with halogenated solvents. The resulting solutions were visually clear and exhibited appropriate tensile properties for electrospinning. Solutions with PCL concentrations below 12% *w*/*v* produced predominantly beaded or droplet-only morphologies, which is indicative of insufficient chain entanglement to sustain a stable Taylor cone and continuous jet. Concentrations above 20% *w*/*v* led to clogging of the needle tip due to rapid solvent evaporation and increased solution viscosity. The usable concentration window was therefore identified as 10–15% *w*/*v*, which is consistent with the values reported for PCL electrospinning from acetic acid-based systems in the literature [24]. Using a combination of parameters in this specified range resulted in the production of membrane samples shown in Figure 1.

### 3.2. Design of Experiments: Optimization of Process Parameters

The DoE analysis identified applied voltage (*A*) and tip-to-collector distance (*B*) as the statistically significant (*p* < 0.05) factors governing mean fiber diameter.

A summary of the ANOVA results for the fitted response surface model is presented in Table 2. The quadratic model exhibited good predictive capability (*R*^2^ = 0.8505, adjusted *R*^2^ = 0.7160) with a non-significant lack of fit (*p* = 0.0636), confirming its adequacy for describing the experimental data within the studied factor space.

An adequate precision ratio of 8.638 (desirable threshold: >4) further confirms that the model provides a reliable signal for navigating the design space.

The empirical model for mean fiber diameter expressed in terms of coded factors takes the following form:

ŷ = 18.21 + 5.12 A + 3.26 B + 1.03 C + 2.88 AB − 0.78 AC − 1.82 BC − 5.12 A^2^ − 4.42 B^2^ + 2.88 C^2^ (1)

where *A* = applied voltage (coded), *B* = tip-to-collector distance (coded), and *C* = flow rate (coded).

The corresponding equation in terms of actual factors is shown below:

ŷ = −45.42 + 6.36 (Power) + 5.96 (Distance) − 4.00 (Flow rate) + 0.142 (Power × Distance) − 0.0497 (Power × Flow rate) − 0.115 (Distance × Flow rate) − 0.253 (Power^2^) − 0.218 (Distance^2^) + 0.235 (Flow rate^2^) (2)

The optimization performed using the desirability function approach, targeting the simultaneous minimization of mean fiber width and height (Figure 1), which identified the following optimal conditions: Power = 8 kV; Distance = 8 cm; Flow rate = 11.42 mL/h (Solution 1, desirability = 0.912; Table 3). Under these conditions, the minimal predicted targeted sample size was 4.387 with an average of 4.535 ± 0.181 cm^2^ (Table 3).

### 3.3. Optimized Green-Solvent Electrospun Fibers: Comparison with PCL and Conventional-Solvent Fibers

#### 3.3.1. Morphology Analysis by Scanning Electron Microscopy

**Figure 2:**
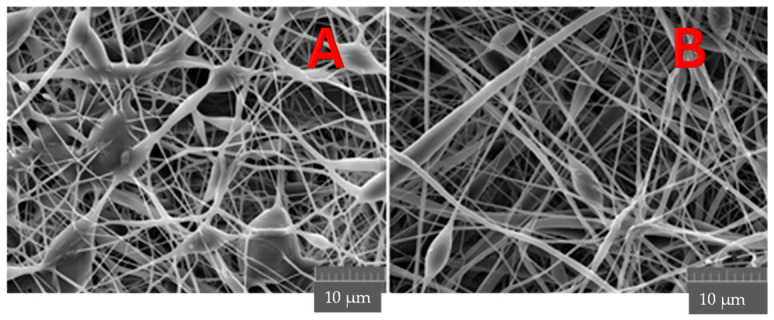
SEM micrographs of electrospun fibers from (**A**) DCM:DMF (8:2) solvent system and (**B**) FA:HAc (1:1) solvent systems.

SEM micrographs are shown in Figure 2. A sample of 12% PCL electrospun from the mixture of DCM:DMF clearly exhibits a higher frequency of fiber–fiber fusion and a significant population of bead-on-string defects. The beads are quite prominent, acting as focal points where multiple fibers coalesce. This indicates that in this specific processing window, the jet is not fully solidifying before reaching the collector, leading to the “wet” deposition that causes fusion and bead formation. This indicates that the conditions used for green solvent were not perfectly optimized for this solvent mixture. The sample from green solvent displays a more uniform, fibrous network with relatively fewer defects compared to the DCM:DMF sample. While there are some spindle-shaped beads present, the fibers are generally more discrete and exhibit less extensive fusion than those in the DCM:DMF sample. This suggests that the green solvent system, under the conditions used, provided a more stable Taylor cone and a more favorable evaporation rate, allowing for better-defined fiber drawing.

The morphological differences of the electrospun matrices induced by the choice of solvent system were evaluated through a comparative statistical analysis of fiber diameters obtained from SEM microphotographs. The statistical distribution reveals a distinct shift toward finer fiber populations when utilizing the sustainable, green solvent mixture of formic acid and acetic acid (FA:HAc (1:1)) compared to the conventional dichloromethane and dimethylformamide system (DCM:DMF (8:2)). The average fiber diameter for the PCL_FA_HAc fibers was 247 nm, marking a substantial 37.3% reduction against the 394 nm average exhibited by the PCL_DCM_DMF matrix. This structural downscaling is highly advantageous for organ-on-a-chip applications, where the electrospun mat serves as a biomimetic membrane or porous barrier separating cellular compartments. Smaller fiber diameters exponentially increase the specific surface-area-to-volume ratio, closely mimicking the ultrastructure of the natural basement membrane, which optimizes cell attachment, barrier integrity, and physiological signaling under microfluidic shear stress.

**Figure 3:**
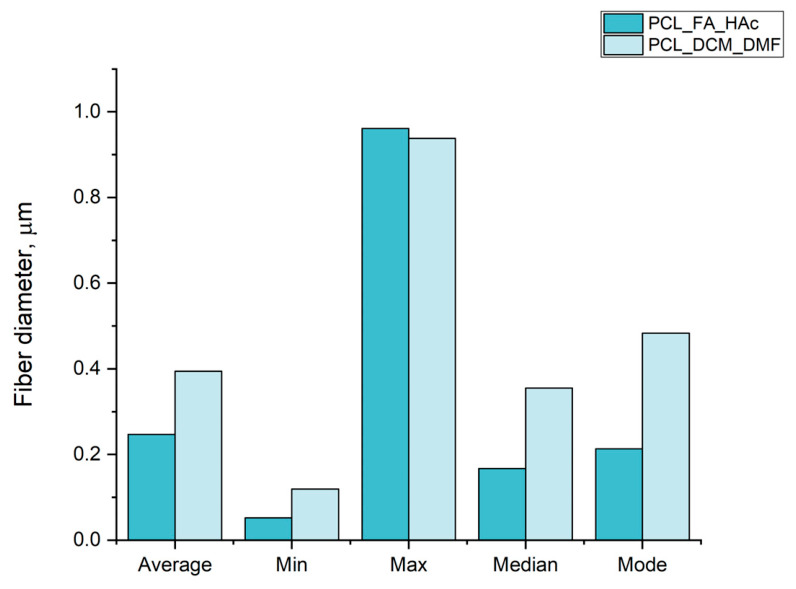
Statistical analysis and comparison of fiber diameter distribution of electrospund fibers from DCM:DMF (8:2) solvent system and FA:HAc (1:1) solvent systems.

A deeper analysis of the positional averages (Figure 3) highlights a right-skewed distribution for the green solvent system, where the median of 167 nm sits considerably lower than the mean, which is driven by an ultra-fine sub-population that reaches a minimum diameter of 52 nm. In contrast, the conventional system favors coarser morphology, yielding a median of 355 nm and a predominant mode peaking at 483 nm. Mechanistically, the capacity of the FA:HAc (1:1) system to yield finer fibers is attributed to the higher dielectric constants and net electrical conductivity intrinsic to organic acids, which elevate the surface charge density of the jet and induce stronger electrostatic repulsive forces and whipping instabilities during elongation, which is in agreement with established electrospinning principles, where increasing the solution’s overall conductivity is known to directly drive down fiber diameters. While the high volatility of DCM drives rapid phase separation, the lower conductivity of the DCM:DMF (8:2) system limits extensive jet stretching, consolidating into thicker fiber segments.

Both systems occasionally exhibit isolated thick fibers near the maximum range (961 nm for FA:HAc and 938 nm for DCM:DMF), and the exceptionally low median and mode of the green formulation confirm that these larger values represent minor outliers within a highly uniform, nanostructured network. However, although the majority of the electrospun fibers exhibited diameters within the expected submicron range, a small fraction of thicker fibers (>900 nm) was observed in both solvent systems. The occurrence of these larger fibers may be attributed to transient instabilities during the electrospinning process, which can lead to fluctuations in jet stretching and consequently result in the formation of fibers with increased diameters.

In addition, environmental conditions, particularly relative humidity, may have influenced fiber formation. Variations in humidity can affect solvent evaporation rates, solution viscosity at the jet surface, and charge dissipation, thereby contributing to local changes in fiber morphology and diameter distribution. Small fluctuations in temperature and airflow within the electrospinning chamber may further enhance these effects.

Another possible explanation is the presence of localized variations in polymer concentration or incomplete homogenization of the spinning solution, which can temporarily increase chain entanglement density and reduce jet elongation. As a result, thicker fibers may be produced intermittently alongside the predominant population of finer fibers. Since such fibers were detected in both systems, their formation appears to be associated primarily with inherent process variability rather than with the specific solvent composition. Nevertheless, the relatively low proportion of fibers exceeding 900 nm indicates that stable electrospinning conditions were generally maintained.

These results demonstrate that transitioning to a green formulation eliminates the risk of toxic chemical leaching into microfluidic channels, which could otherwise compromise delicate cellular assays on-chip, and it also actively optimizes the structural architecture of the membrane by driving fiber dimensions down to the true nanoscale.

#### 3.3.2. FTIR Spectroscopic Analysis

**Figure 4:**
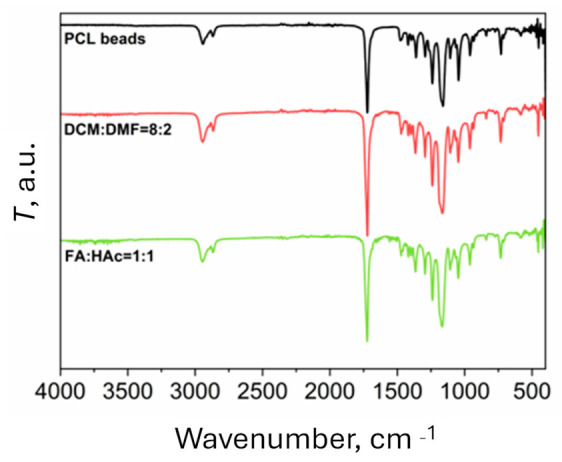
FTIR-ATR spectra of the PCL and the electrospun membranes.

FTIR-ATR spectra of the PCL and the electrospun membranes are compared in Figure 4.

As can be seen in Figure 4, both spectra display the characteristic absorption bands of PCL: the strong carbonyl stretching vibration (C=O) at ~1722 cm^−1^, C–O–C asymmetric stretching at ~1238 cm^−1^, and C–H stretching bands in the region 2940–2860 cm^−1^. Importantly, no new absorption bands attributable to acetic or formic acid residues (e.g., carboxylic O–H stretch at 2500–3300 cm^−1^ or form C–H at ~2720 cm^−1^) were detected in the membrane spectra, confirming efficient solvent evaporation during the electrospinning process and the absence of solvent contamination in the final membrane. The chemical integrity of PCL was thus fully preserved, which is a prerequisite for the biocompatibility of material in cell culture applications.

#### 3.3.3. Diffraction Analysis

X-ray diffraction was performed to assess the structural differences between PCL and fibers electrospun from conventional and green solvent systems (Figure 4). It is evident from the patterns that all samples exhibit diffraction maxima typical to that of crystalline PCL with some differences depending on the synthesis route. The PCL crystalline structure can best be confirmed by the presence of peaks at ~21.3° and ~23.6°, which correspond to the (110) and (200) plane, respectively. By examining the peak width, it is evident that electrospun fibers show a more defined crystalline structure than the PCL beads. The appearance of additional diffraction maxima, such as the shoulder peak at ~21.9°, which belongs to the (110) crystal plane, further shows the more defined crystalline structure after electrospinning. The overall higher intensity of fibers electrospun from a mixture of DCM and DMF does not necessarily indicate higher crystallinity, as the membrane thickness is not equal in both cases, but the increase in sharpness of the diffraction maxima at ~16.9° indicates that the structural order is increased. Regardless of the significantly higher volatility of DCM, whose boiling point is significantly lower (~39 °C) than the boiling points of all the other solvents (~153, 101 and 118 °C for DMF, FA and Hac, respectively), the DCM:DMF = 8:2 fibers result in higher crystallinity. Considering the drying time profiles, it can be observed that the FA:Hac = 1:1 solution will dry relatively evenly due to the similar boiling points of the solvents, while the DCM:DMF = 8:2 solution evaporates unevenly, with DCM leaving the solution almost instantaneously and DMF remaining in the system, possibly even after the fibers have reached the substrate. The residual DMF concentration is not high enough to collapse the whole structure by dissolving the electrospun fibers, but it is enough to prolong the drying time in atmospheric conditions, which allows for additional ordering and increased crystallinity.

**Figure 5:**
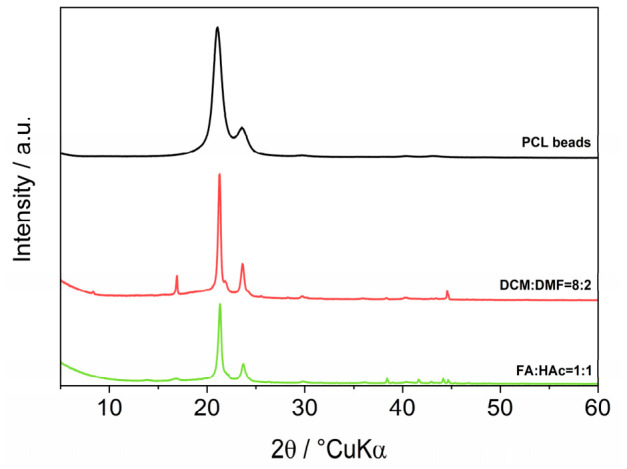
Diffraction patterns of the samples.

An interesting observation that also points to this is the appearance of a very small, peculiar peak at ~8.3° which corresponds to a d-spacing of 1.06 nm (Figure 5). Considering that structural information such as this is not common in the literature, it is unclear as to what this peak belongs to exactly, but considering the X-ray scattering data published on these materials, this peak most likely belongs to the ordering of lamellae formations in the polymer, as the scattering vector (q) for this peak corresponds to 0.59 Å^−1^, which falls within the range where scattering is usually employed to study polymers (q = 0–2 Å^−1^), implying an additional architectural ordering in fibers when compared to the bulk polymer [31]. From these results, it can be strongly assumed that the high-velocity flow-induced alignment of the chains in the polymer solution prior to ejection promotes increased chain alignment in the fibers, which increases supramolecular stacking and results in higher overall crystallinity despite the rapid solvent evaporation.

The observed increase in crystal ordering, together with the emergence of more ordered lamellar structures in the electrospun polymer samples compared to raw PCL, surely contributes to a variety of properties, including thermal stability and mechanical properties which are discussed in the following sections.

#### 3.3.4. Thermal Analysis

**Figure 6:**
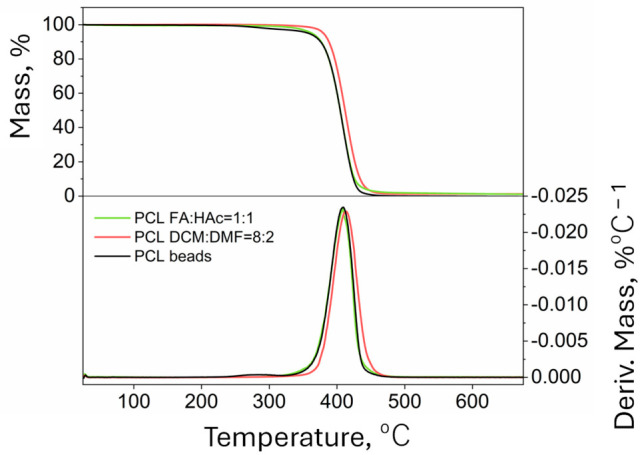
TG and DTG curves of the samples.

Thermograms (Figure 6, Table 4) confirm a one-step degradation process for both electrospun samples, which is governed by specific chain scission, typically occurring between 300 °C and 500 °C. Raw PCL additionally shows a lower temperature degradation process (evident also from DTG), which can be attributed to random chain scission, which is a process dominant at temperatures below 300 °C. Electrospun samples show negligible weight loss (<0.55 mass. %) up to 160 °C with less than 0.05 mass. % in the 100–160 °C temperature range where DMF, acetic and formic acid evaporate, confirming findings from FTIR that there is no residual solvent in either sample.

The thermal stability of PCL is significantly enhanced through the electrospinning process, as evidenced by the increase in the onset of degradation and *T*_5%_ temperatures compared to PCL beads. This enhancement is likely driven by increases in crystallinity and polymer chain alignment induced during fiber formation. Regardless of the small variations in crystallinity between the electrospun samples, the thermal analysis correlates to structural analysis very well, as significant differences in the onset degradation temperature (taken at 5% mass loss) may be observed for all three samples.

The difference in thermal stability between raw PCL (onset at 356 °C) and electrospun fibers (onset at 364 and 380 °C) stems from the overall increased crystallinity, which is evident by the increased sharpness of peaks and the appearance of additional reflections. Following the same logic, the long-range order that the DCM:DMF = 8:2 sample possesses, such as the peaks at 16.9° and 8.3°, which correspond to lamellae formations, may further explain the discrepancy between the onset degradation temperature between the two electrospun fibers (364 and 380 °C).

**Figure 7:**
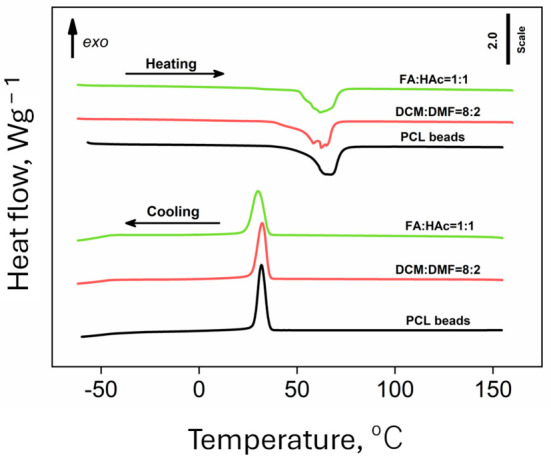
DSC thermograms of the samples.

The differential scanning calorimetry (DSC) analysis reveals certain variations in the thermal transitions of PCL following different processing routes (Figure 7). The PCL beads exhibit a slightly higher melting enthalpy than the electrospun fibers, indicating a higher yield of crystalline phases. These findings corroborate the structural reorganization observed via XRD, indicating that while the raw material possesses higher absolute enthalpy and more, the electrospun scaffolds feature a more refined and thermally stable crystalline morphology. It can also be observed that the melting point of the electrospun fibers is lower than that of the raw material, which could be the consequence of the significantly increased surface area of the fibers, which lowers the energy required for phase transition. However, once the fibers are melted, the crystallization peak is practically identical as expected.

The sample electrospun from green solvents shows comparative properties to the sample electrospun from conventional solvents, thus confirming the viability of this solvent system for PCL fiber electrospinning (Figure 7).

The only shortcomings of the green synthesized sample are slightly reduced melting point and according to the melting enthalpy, a reduced yield of polymer crystallinity.

This also correlates well to the increased degradation onset temperature (taken at 5% mass loss) for both electrospun fibers, as an increased degree of crystallinity reduces polymer backbone mobility, which significantly increases the kinetic energy required for random scission and suppresses the process entirely (Table 4).

#### 3.3.5. Tensile Tests

**Figure 8:**
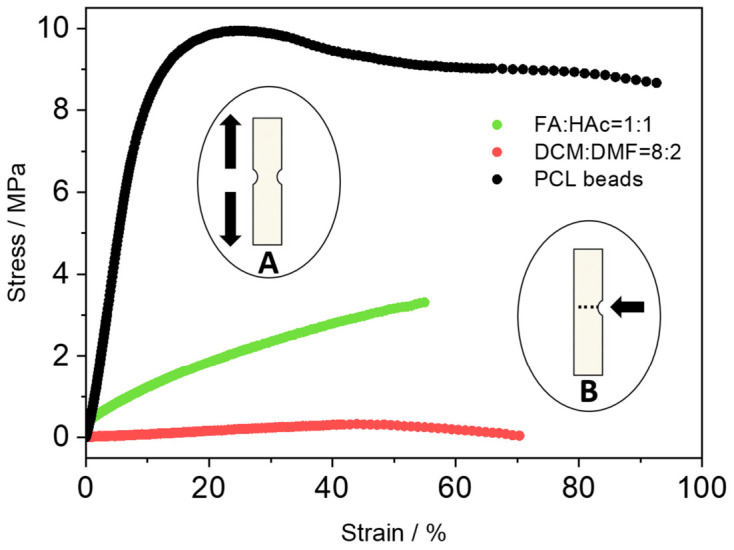
Tensile tests performed on the PCL beads and electrospun fibers with (**A**) a schematic representation of sample breakage for the PCL beads and FA:HAc sample and (**B**) schematic representation of sample breakage for the DCM:DMF sample.

Mechanical testing was utilized to assess the materials’ Young modulus and elongation, whose results are given in Table 5 and shown in Figure 8. Foils pressed from raw PCL show the highest Young modulus comparable to those in the literature. What is interesting is that there is an order of magnitude difference between the stiffness of FA:HAc = 1:1 and DCM:DMF = 8:2 with the FA:HAc being the one with a higher Young modulus. Such a large difference could stem from a difference in porosity, where the DCM:DMF sample has a higher specific surface area which compromises its mechanical properties. It can also be observed that the DCM:DMF sample possesses a greater concentration of beads which are known to be weak spots for mechanical stress and can contribute to the degradation of mechanical properties [32]. When considering fracture strain, the materials act relatively similarly with FA:HAc having somewhat higher elongation at fracture despite the DCM:DMF giving higher apparent elongations. This is because the DCM:DMF sample did not break “correctly” in the middle of the sample but rather started ripping from one side to the other, which causes the breakage curve to go down slowly (Figure 8B) instead of abruptly ending like it does for the other two samples (Figure 8A).

These findings highlight the strong relationship between processing parameters, scaffold morphology, and mechanical behavior, demonstrating that even subtle variations in electrospinning conditions can significantly alter the structural integrity and performance of electrospun PCL materials. Consequently, a careful optimization of processing parameters is essential and should be tailored to the specific solvent system employed, as differences in solvent properties can profoundly influence fiber morphology and, in turn, the resulting mechanical properties.

### 3.4. Suitability for Organ-on-a-Chip and Multi-Layer Applications

The optimized PCL membranes satisfy several critical criteria for integration into OoC platforms. Further functionality can be added by integrating the membrane with on-chip sensing elements—for example, flexible inkjet-printed pH sensors have recently been demonstrated for real-time biomedical monitoring within OoC environments, underscoring the growing convergence of materials science, microfluidics, and embedded sensing in these platforms [33].

The mechanical flexibility of PCL membranes is an additional asset in OoC contexts, particularly in systems that incorporate cyclic mechanical stimulation (e.g., breathing motion in lung-on-a-chip, or peristalsis in gut-on-a-chip) via pneumatic actuation. PCL’s low Young’s modulus of 7–13 MPa (for electrospun fibers) closely matches the compliance requirements of such dynamic culture systems, and its long degradation timescale ensures membrane integrity over the typical duration of OoC experiments (days to weeks) [34].

Beyond single-membrane OoC applications, the membranes can serve as building blocks in hierarchical multi-layer constructs. By sequentially stacking or bonding membranes of differing fiber diameters, orientations, or surface chemistries, it is possible to engineer anisotropic scaffolds that replicate the lamellar organization of tissues such as cornea, myocardium, or tendon [35,36]. This concept aligns with recent work demonstrating a sustainable net-zero production of advanced biopolymer membranes for analogous biomedical purposes, further supporting the green-chemistry rationale of this paper [37,38,39]. The demonstrated process scalability—enabled by the safe and widely available green solvents—supports the potential for the translational manufacturing of such advanced constructs at both laboratory and industrial scale [40,41].

The elimination of DMF and DCM from the PCL electrospinning process represents a meaningful contribution to the broader effort of harmonizing biomedical materials research with the imperatives of sustainable chemistry [42]. From a regulatory standpoint, the use of acetic and formic acid is expected to simplify the approval pathway for investigational devices incorporating these membranes, as residual solvent limits for these compounds (ICH Q3C Class 3) are substantially more permissive than those for DMF (Class 2) or DCM (Class 2) [43].

The regulatory landscape has also shifted decisively in favor of OoC-based evidence. The FDA Modernization Act 2.0 removed the statutory requirement for animal testing, while the FDA’s ISTAND Program and its 2025 Roadmap create concrete qualification pathways for OoC devices as drug development tools [3,5,6,44]. The GAO’s 2025 report (GAO-25-107335) identifies standardized, validated membranes with well-defined physicochemical properties as a prerequisite for the regulatory acceptance of OoC data packages—which is a requirement directly addressed by the systematic DoE-based characterization approach presented here [4,7].

Therefore, the materials developed in this paper satisfy the criteria for OoC materials in several aspects. First, the nanofibrous architecture mimics the structural features of the native ECM, providing topographical cues that promote cell adhesion and directed morphogenesis—without the need for additional surface functionalization, which is often required for flat polymeric membranes. Second, the controlled porosity allows for selective molecular transport while maintaining cellular compartmentalization, enabling the co-culture of different cell types on opposing membrane surfaces, as is required in gut-on-a-chip or lung-on-a-chip configurations [45,46].

The methodology enabled an efficient characterization of novel membranes. FTIR analysis was performed to assess the structural differences between PCL and fiber electrospun from conventional and green solvent systems (Figure 4). It is evident from the patterns that all samples exhibit diffraction maxima typical to that of crystalline PCL with some differences depending on the synthesis route. The PCL crystalline structure can best be confirmed by the presence of peaks at ~21.° and ~23.6°, which correspond to the (110) and (200) plane, respectively [47]. By examining the peak width, it is evident that electrospun fibers show a more defined crystalline structure than the PCL beads.

Thermograms (Figure 6, Table 4) confirm a one-step degradation process for both electrospun samples, which is governed by specific chain scission, typically occurring between 300 °C and 500 °C. Raw PCL additionally shows a lower temperature degradation process (evident also from DTG) that can be attributed to random chain scission, which is a process dominant at temperatures below 300 °C [48]. Electrospun samples show negligible weight loss (<0.55 mass. %) up to 160 °C with less than 0.05 mass. % in the 100–160 °C temperature range where DMF, acetic and formic acid evaporate, confirming findings from FTIR that there is no residual solvent in either sample.

Mechanical testing was utilized to assess the materials’ Young modulus and elongation whose results are given in Table 5 and shown in Figure 8. Foils pressed from raw PCL show the highest Young modulus, comparable to those in the literature, while the electrospun fibers exhibit lower values like those published by Croisier et al. where they measured the Young modulus of electrospun fiber scaffolds to be 3.8 ± 0.8 MPa [49]. What is interesting is that there is an order of magnitude difference between the stiffness of FA:HAc = 1:1 and DCM:DMF = 8:2 with the FA:HAc being the one with a higher Young modulus. Such a large difference could stem from a difference in porosity, where the DCM:DMF sample has a higher specific surface area which compromises its mechanical properties. It can also be observed that the DCM:DMF sample possesses a greater concentration of beads which are known to be weak spots for mechanical stress and can contribute to the degradation of mechanical properties [32]. When considering fracture strain, the materials act relatively similarly with FA:HAc having somewhat higher elongation at fracture despite the DCM:DMF giving higher apparent elongations. This is because the DCM:DMF sample did not break “correctly” in the middle of the sample but rather started ripping from one side to the other, which causes the breakage curve to go down slowly (Figure 8B) instead of abruptly ending like it does for the other two samples (Figure 8A).

## 4. Conclusions

This paper demonstrates the successful development of a fully green, sustainable binary solvent system comprising acetic acid and formic acid for the electrospinning of polycaprolactone (PCL) membranes targeted at organ-on-a-chip (OoC) platforms. Based on the systematic experimental design and multi-technique characterization, the following key conclusions can be drawn (i) Green Process Viability: PCL dissolves readily in bio-derived acetic acid/formic acid mixtures at room temperature, offering an environmentally friendly alternative that completely eliminates the reliance on hazardous, REACH-regulated organic solvents such as DCM and DMF (ii) Morphological Optimization: Under optimized Design of Experiments (DoE) parameters, the green solvent formulation yields uniform, continuous, and highly discrete nanofibers. The resulting mean fiber diameter of 247 nm represents a substantial 37.3% reduction compared to conventional DCM:DMF-spun matrices, providing an ultra-fine, biomimetic architecture that maximizes specific surface area (iii) Chemical and Structural Integrity: FTIR-ATR analysis confirmed the total evaporation of volatile organic acids, leaving zero residual solvent contamination and maintaining the full chemical integrity of the native polymer. XRD, DSC, and TGA profiles revealed a highly ordered lamellar arrangement and excellent thermal stability (onset degradation at 364 °C), validating the structural viability of the green-spun scaffolds. (iv) Suitable Mechanical Properties: Robust mechanical compliance (Young’s modulus of 7–13 MPa) mimics native extracellular matrix basements, making the membranes ideal for integration into dynamically actuated microphysiological devices.

Future work will focus on the in vitro cytocompatibility assessment of the membranes with relevant cell lines, the integration of the optimized membranes into prototype microfluidic devices, and the exploration of surface modification strategies (e.g., plasma treatment, collagen coating) to further enhance cell attachment and functionality within the OoC context.

## Figures and Tables

**Table 1:** Design of Experiments factor levels and experimental ranges.

Factor	Symbol	Low (−1)	Centre (0)	High (+1)
Applied voltage (kV)	A	8	13.50	17
Flow rate (mL/h)	B	0.10	0.81	1.7
Tip-to-collector distance (cm)	C	8	13.50	17

**Table 2:** ANOVA summary for the response surface quadratic model of mean fiber diameter (*n* = 20 runs). SS, sum of squares; df, degrees of freedom; MS, mean square. Significant model terms are indicated in bold.

Source	SS	df	MS	F-Value	*p*-Value	
**Model**	**736.43**	**9**	**81.83**	**6.32**	**0.0040**	**significant**
A—Power	243.52	1	243.52	18.82	0.0015	
B—Distance	106.71	1	106.71	8.25	0.0166	
C—Flow rate	10.72	1	10.72	0.83	0.3842	
AB	46.35	1	46.35	3.58	0.0877	
AC	3.37	1	3.37	0.26	0.6209	
BC	21.64	1	21.64	1.67	0.2250	
A^2^	107.89	1	107.89	8.34	0.0162	
B^2^	69.40	1	69.40	5.36	0.0431	
C^2^	33.58	1	33.58	2.59	0.1383	
Residual	129.42	10	12.94			
Lack of fit	105.66	5	21.13	4.45	0.0636	not significant
Pure error	23.76	5	4.75			
**Cor Total**	**865.84**	**19**				

R^2^ = 0.8505; Adj. R^2^ = 0.7160; Pred. R^2^ = 0.0001; Adeq. Precision = 8.638; C.V. = 25.57%.

**Table 3:** Top-ranked solutions from the desirability-function multi-response optimization (minimize fiber width and height). The selected solution is presented in bold letters.

No.	Power (kV)	Distance (cm)	Flow Rate × 10^−3^ (mL/h)	Width (cm)	Height (cm)	Sample Size ** (cm^2^)
1 *	**8.000**	**8.000**	**11.421**	**2.019**	**2.173**	4.387
2	8.000	8.000	11.484	2.024	2.170	4.392
3	8.000	8.000	11.357	2.017	2.178	4.393
4	8.000	8.000	11.293	2.016	2.184	4.403
5	8.000	8.000	11.577	2.034	2.169	4.411
6	8.000	8.000	11.223	2.017	2.194	4.425
7	8.004	8.000	10.988	2.051	2.252	4.619
8	8.000	8.000	11.995	2.130	2.213	4.713
9	8.000	8.039	11.496	2.114	2.271	4.800
10	8.000	8.000	10.772	2.081	2.308	4.803

* Selected solution. ** sample size was calculated by multiplying width and height.

**Table 4:** Parameters extracted from the DSC and TGA curves.

Sample	TGA		DSC			
	*T*_5%_/°C	DTG (max.)/°C	Δ*H*_m_/Jg^−1^	Δ*H*c/Jg^−1^	*T*_m_/°C	*T*_c_/°C
PCL beads	356	413	100.2	80.2	66.6	32.1
DCM:DMF = 8:2	380	415	89.8	78.5	62.1	32.7
FA:Hac = 1:1	364	411	88.2	75.9	61.4	30.7

**Table 5:** Young modulus and elongation before breakage.

Sample	Young Modulus/MPa	Fracture Strain/%
PCL beads	70–80	>200
FA:HAc = 1:1	7–13	55–60
DCM:DMF = 8:2	0.6–0.8	45–75

## Data Availability

The data presented in this paper are available on request from the corresponding author.
